# ASOptimizer: optimizing chemical diversity of antisense oligonucleotides through deep learning

**DOI:** 10.1093/nar/gkaf392

**Published:** 2025-05-16

**Authors:** Seokjun Kang, Daehwan Lee, Gyeongjo Hwang, Kiwon Lee, Mingeun Kang

**Affiliations:** Spidercore Inc., 1662, Yuseong-daero, Yuseong-gu, Daejeon 34054, South Korea; Spidercore Inc., 1662, Yuseong-daero, Yuseong-gu, Daejeon 34054, South Korea; Spidercore Inc., 1662, Yuseong-daero, Yuseong-gu, Daejeon 34054, South Korea; Spidercore Inc., 1662, Yuseong-daero, Yuseong-gu, Daejeon 34054, South Korea; Spidercore Inc., 1662, Yuseong-daero, Yuseong-gu, Daejeon 34054, South Korea; Korea Advanced Institute of Science and Technology, 291 Daehak-ro, Yuseong-gu, Daejeon 34141, South Korea

## Abstract

Antisense oligonucleotides (ASOs) are a promising class of gene therapies that can modulate the gene expression. However, designing ASOs manually is resource-intensive and time-consuming. To address this, we introduce a user-friendly web server for ASOptimizer, a deep learning-based computational framework for optimizing ASO sequences and chemical modifications. Given a user-provided ASO sequence, the web server systematically explores modification sites within the nucleic acid and returns a ranked list of promising modification patterns. With an intuitive interface requiring no expertise in deep learning tools, the platform makes ASOptimizer easily accessible to the broader research community. The web server is freely available at https://asoptimizer.s-core.ai/.

## Graphical Abstract

**Graphical Abstract:**
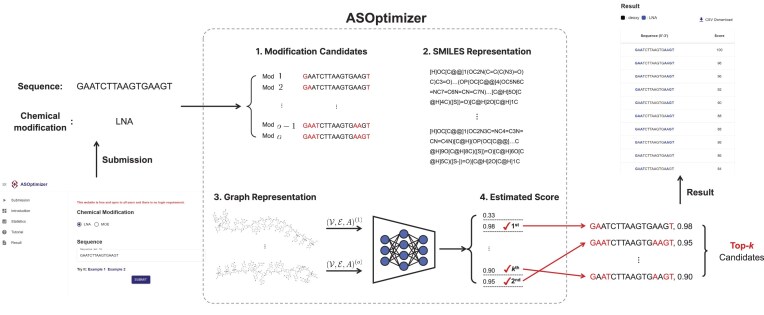

## Introduction

Antisense oligonucleotides (ASOs) are short single-stranded synthetic nucleotides that bind to target RNA through complementary base pairing [1–3]. They serve as an effective platform for a wide range of rare and genetic diseases, including Duchenne muscular dystrophy, spinal muscular atrophy, and hereditary transthyretin amyloidosis [4–8]. However, designing ASOs remains a challenging problem due to the vast number of possible combinations. For example, an ASO of length *l* has 4^*l*^ sequence variations, as each nucleotide can be A, U/T, G, or C. In addition, various chemical modifications have been introduced to improve ASO stability and binding affinity [9–12], further increasing the complexity of the design process.

For simplicity, researchers typically rely on candidate sequences that are complementary to the target RNA and adopt the gapmer architecture [11]. Gapmer ASOs feature a central segment of DNA bases flanked by chemically modified RNA bases at both ends [13]. This architecture is widely used for RNase H-mediated gene regulation, offering improved knockdown activity and reduced cytotoxicity than plain phosphorothioate (PS) ASOs. However, recent studies have shown that standard gapmers often lead to suboptimal therapeutic performance, including reduced inhibition efficacy and increased cytotoxicity [14–17]. These observations raise the question of how ASOs can be systematically designed *in silico* for optimal performance.

Extensive studies have been conducted to investigate the relationship between therapeutic RNA candidates and their efficacy. Depending on RNA modality, they can be categorized into (i) Small interfering RNA (siRNA)-related and (ii) exon-skipping ASO-related approaches. For siRNAs, researchers have identified early protocols for improved siRNA efficacy, such as low G/C content, avoidance of a long G/C stretch, and the presence of TT overhangs at 3' ends [18–21], which later led to off-target-related factors [22, 23]. These findings led to the development of efficacy prediction algorithms, which now serve as the foundation for siRNA design tools [24–28]. Similarly, for exon-skipping ASOs, critical design factors have been identified [29, 30], enabling the development of predictive models for efficacy [31, 32]. Despite these advancements and available software [33–35], a major limitation remains: current methods do not optimize the chemical modifications of therapeutic RNAs.

We recently introduced ASOptimizer [36], the first computational method for optimizing ASOs at the molecular level. The optimization involves a novel mathematical problem that can be efficiently solved via deep learning. Until now, ASOptimizer has been available only as a Python-based GitHub repository, requiring users to have familiarity with modern deep learning libraries. To make ASOptimizer more accessible, we present a web server that can broaden its applications. The platform features an intuitively designed, user-friendly interface and provides graphics processing unit (GPU) resources in the back end. Hence, researchers and industry professionals—regardless of their background in deep learning—can easily obtain potent ASO candidates. This will significantly reduce the time and resources required for gapmer ASO development, contributing to faster drug development and broader adoption of ASOs in gene therapies.

## Method overview

**Figure 1.**
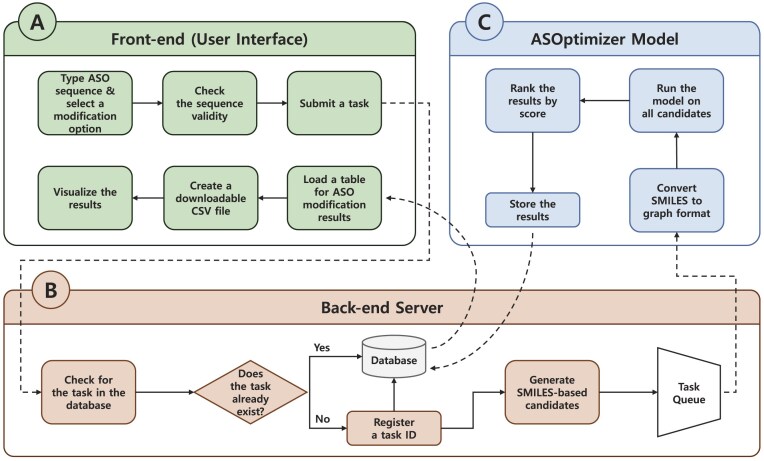
ASOptimizer web workflow. The workflow proceeds through these stages: A (user input via front end) → B (back-end data preprocess and task management) → C (ASOptimizer model inference) → B (result storage in database) → A (display results to user via front end).

ASOptimizer web provides a ranked list of chemically modified ASOs based on predicted performance, given a user-submitted sequence. To this end, we developed a web service architecture consisting of front-end and back-end servers that communicate through a REST API (see Fig. 1). The front-end server receives an ASO sequence and a selected modification type from the user and transmits this information to the back end after validating the input. The back-end server scans the hash table database for redundancy; if no matching ASO sequence is found, it proceeds with a predictive analysis. This analysis begins by generating the simplified molecular-input line-entry system (SMILES) representation and constructing its corresponding graph structure. The graph is then fed into a deep learning model trained to predict ASO efficacy. The results are stored in the hash table to enable reuse when the same query is submitted in the future. Finally, the user receives the output via the front end, which can also be downloaded as a comma-separated value (CSV) file.

The predictions provided by ASOptimizer web are powered by a deep graph neural network (GNN). The model was trained using the same database from our previous study [36], which includes 194 519 quantitative reverse transcription polymerase chain reaction experiments targeting 67 messenger RNAs (mRNAs) and comprising 101 169 unique ASOs. We created SMILES representations of the ASOs using ChemDraw, which were then converted into molecular graphs using RDKit. Here, a molecular graph $\mathcal {G}$ is defined as a pair of two sets: $\mathcal {G} = (\mathcal {V}, \mathcal {E})$, where $\mathcal {V}$ denotes the set of vertices (atoms in the ASO) and $\mathcal {E} \subseteq \mathcal {V} \times \mathcal {V}$ denotes the set of edges (bonds between atoms). These edges can also be presented as an adjacency matrix *A* ∈ {0, 1}^*N* × *N*^, where *A*_*ij*_ = 1 if atoms *i* and *j* are bonded, and *A*_*ij*_ = 0 otherwise. The final dataset $\mathcal {D}$ consists of graph-efficacy pairs: $\mathcal {D} = \lbrace ( x^{(i)}, y^{(i)} ) \rbrace _{i=1}^{m}$, where $x^{(i)} = (\mathcal {V}^{(i)}, \mathcal {E}^{(i)}, A^{(i)})$ represents the molecular graph of the *i*th ASO and *y*^(*i*)^ is its experimental inhibition rate.

For the network architecture, we adopted edge-augmented graph transformer (EGT) [37], a state-of-the-art GNN for multiple graph-related tasks. We trained the EGT model in a learning-to-rank fashion [38], as the dataset includes varying conditions—such as cell lines and dosages—that can affect knockdown efficacy. Specifically, our model *f*_*θ*_, parameterized by *θ*, compares two samples *x*_pos_ and *x*_neg_ at a time. Each pair shares a common nucleotide sequence and experimental conditions but differs in chemical modifications. The parameter *θ* was optimized via RMSProp [39], minimizing the objective:


(1) \begin{eqnarray*}
\theta ^{*} = \arg \min _{\theta } \sum _{i=1}^{m_{\text{pairs}}} \max (0, t + f_{\theta }(x_{\text{neg}}^{(i)}) - f_{\theta }(x_{\text{pos}}^{(i)})) .
\end{eqnarray*}

To reduce inference latency in the web environment, the model was trained using 16-bit mixed precision. Other hyperparameters related to the model architecture and training are summarized in Supplementary Table S1.

Given a user-submitted ASO sequence and a selected chemical modification type, the back-end server generates combinations of modification patterns using PS and the specified modification. The computing server, equipped with GPU capabilities, receives the graph representations of the generated patterns and sequentially predicts their inhibition rates. As this process is computationally intensive due to heavy GPU operations, we implemented a task queue system to efficiently manage resources and streamline the service. The queue dynamically adjusts the number of concurrent tasks based on server load. It also enhances user experience by providing a real-time progress bar and task IDs, allowing users to re-access previous jobs even after closing the webpage.

## User interface and input/output description

The ASOptimizer web server offers a user-friendly interface, structured with a submission page, a loading page, a result page, and a downloadable CSV results file.

**Figure 2.**
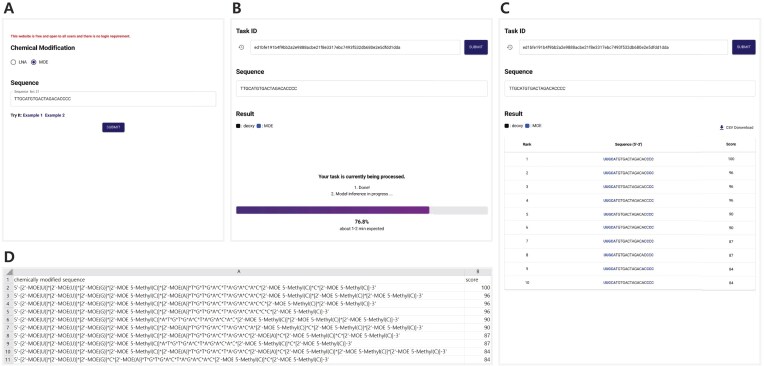
ASOptimizer web server user interface. (**A**) Submission page with input fields for ASO sequence and modification type selection. (**B**) Loading page with task ID. (**C**) Result page featuring the task ID, prediction result table, and CSV download button. (**D**) Format of the downloaded CSV results file.

The submission page (Fig. 2A) serves as a primary interface where users type or paste their ASO sequence to initiate the optimization process. The input must be a string composed of A, T, C, and G, with a maximum length of 22 bases; only one sequence can be submitted at a time. This length constraint ensures efficient resource usage and rapid analysis in the web environment. Users can also select the desired chemical modification type using a drop-down menu. Currently, the ASOptimizer web server supports a single modification type, where each base is modified with either PS or the selected one. Available options include MOE (2'-*O*-methoxyethyl) and LNA (locked nucleic acid). Upon submission, the web server initiates the analysis. A unique task ID is displayed at the top of the loading page (see Fig. 2B), allowing users to monitor progress and revisit results later.

Once inference in the computing server is complete, the user interface automatically transitions to the result page (Fig. 2C). This page shows the user-submitted input and the corresponding task ID. The core output is displayed as a table listing the top 10 modification patterns, ranked by their predicted scores. Each row highlights the modification positions in blue along with the predicted score for that pattern. A higher score indicates greater predicted efficacy. Users can download the full prediction results in CSV format by clicking the download button located at the top of the table.

The CSV file (Fig. 2D), downloaded as [sequence]-[chemical modification].csv, provides a structured output for downstream analysis. It contains two columns: “chemically modified sequence” and “score.” The sequence column displays nucleotide sequences, with chemical modifications represented according to the conversion rules detailed in Table 1. Within this column, an asterisk symbol (*) indicates a PS linkage between nucleotides. The score column presents the associated prediction score generated by the model. Note that the results are ranked in descending order of score.

**Table 1.** Conversion rules for chemically modified sequences in output CSV

Modification	DNA base	Modified base
MOE	T	[2’-MOE(U)]
	C	[2’-MOE 5-Methyl(C)]
	A	[2’-MOE(A)]
	G	[2’-MOE(G)]
LNA	T	[LNA T]
	C	[LNA C]
	A	[LNA A]
	G	[LNA G]

## Web implementation

The ASOptimizer web server was designed to maximize efficient use of available computing resources. Both the front-end and back-end servers run on Intel^®^ Xeon^®^ Silver 4114 CPU @ 2.20 GHz, and the back-end server is additionally equipped with two NVIDIA TITAN RTX GPUs to support deep learning inference. For the back-end framework, we adopted a Python-based Flask, which offers scalability, fast execution, and seamless integration with deep learning and bioinformatics libraries such as TensorFlow [40] and RDKit. The front end was developed using React and Vite; React’s component-based architecture simplifies maintenance and scaling, while Vite enables fast, lightweight builds for an enhanced user experience. A significant portion of error handling was also implemented on the front end to reduce computational load and potential bottlenecks on the back-end server.

To further improve GPU utilization, we incorporated the Celery task queue system. Celery dynamically adjusts the number of concurrent tasks based on server load, ensuring stable performance even under constrained resources. Communication between the front-end and back-end servers is handled via a REST API, employing a polling mechanism to minimize unnecessary connections. The progress and result data of each task are managed using a PostgreSQL database. A unique task ID is assigned for each request and stored in the database, allowing users to revisit and retrieve their results at any time—even after closing the webpage.

## Results

To validate ASOptimizer, we tested the model on additional datasets composed of unseen examples. Performance was evaluated using the Pearson correlation coefficient (*ρ*) between the predicted outcomes and the corresponding experimental inhibition rates.

### Comparison with patent data

**Figure 3.**
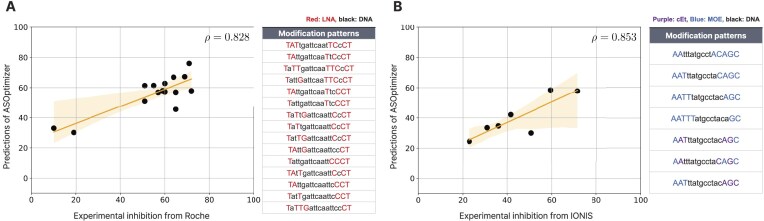
*In silico* validation on external patent data. (**A**) Scatter plot of prediction scores produced by ASOptimizer and experimental inhibition rates on ASOs with LNA/DNA architecture. (**B**) Scatter plot similar to the left on ASOs with cEt/MOE/DNA architecture. Tables indicate which patterns are used for evaluation.

Fig. 3A shows a scatter plot of 15 modification patterns of LNA/DNA for the ASO 5′-TATTGATTCAATTCCCT-3′, with experimental data obtained from a Roche patent titled Oligonucleotides for Inducing Paternal Ube3a Expression. We observed substantial variation in inhibition rates for the same sequence depending on the modification pattern. ASOptimizer achieved a strong correlation of *ρ* = 0.83 with the experimental results.

Figure 3B is a scatter plot on seven patterns of cEt/MOE/DNA modifications for the ASO 5′-AATTTATGCCTACAGC-3′, with experimental data obtained from an IONIS patent titled Modulation of hepatitis B virus (HBV) expression. Similar to the previous case, knockdown efficacy varied across patterns, highlighting the importance of modification patterns. ASOptimizer achieved a correlation of *ρ* = 0.85.

### Comparison with [16] on LNA modification

**Figure 4.**
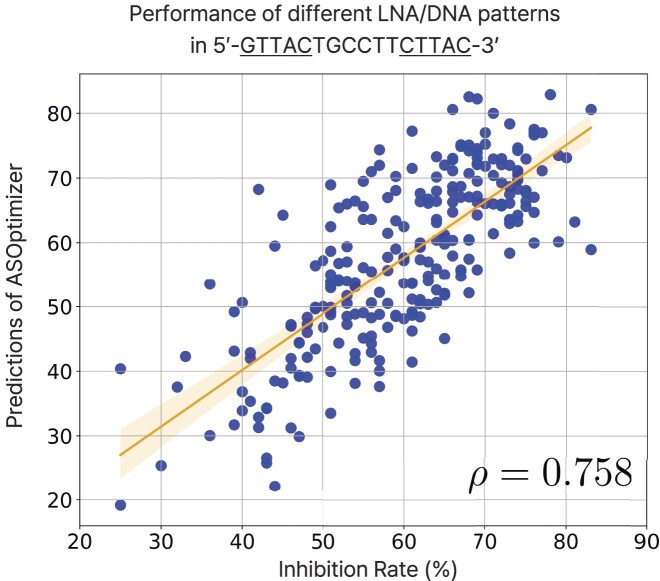
Scatter plot of measured (experimental) HIF1A mRNA inhibition rate [16] versus inhibition score estimated by ASOptimizer.

We further evaluated the model using DNA-versus-LNA combinations. Following the design rule in [16], we generated 2^4^ = 16 modification cases for each wing of five nucleotides, changing positions 2–5 in each wing. This resulted in a total of 256 distinct LNA modification patterns per sequence. As the target, we selected a specific region within the hypoxia-inducible factor 1-alpha (HIF1A) mRNA, which is known to be accessible to ASOs with the sequence 5′-GTTACTGCCTTCTTAC-3′. Experimental inhibition data were obtained from [16]. Figure 4 shows a scatter plot of the 256 modification architectures. ASOptimizer achieved a correlation of *ρ* = 0.758. Additional *in silico* validation analyses on a broader set of ASOs and gene targets are provided in Supplementary Fig. S1.

## Future update plans

ASOptimizer will have major updates that focus on the following features. First, we plan to incorporate sequence-level off-target assessment using tools such as BLAST and Gibbs free energy calculations to evaluate binding potential with non-target transcripts. Second, the deep learning model will be extended to predict the toxicity profiles of the input ASO. Specifically, hepatotoxicity (e.g. ALT and AST) and neurotoxicity performance could be used as measures of toxicity risk, which we already have curated ∼6000 publicly available rodent experiment data. This feature will enable the integration of toxicity filtering into the design pipeline as a post-processing step. Lastly, more diverse chemical modification patterns will be supported beyond MOE and LNA modifications. This extension is feasible due to the presence of rich chemical variants in the training dataset. The update will also include support for mixed modifications, such as 2'-*O*-methyl (2'-OMe) and 2'-fluoro (2'-F), which are frequently used in combination in practice.

## Conclusion

We developed ASOptimizer web, a user-friendly platform that brings our deep learning-based ASO optimization method to the broader research community. The web server is hosted on a GPU-enabled system and currently supports ASOs of up to 22 bases in length with LNA and MOE modifications. The interface is intuitive and freely accessible, offering valuable insights without requiring expertise in deep learning or cheminformatics.

## Supplementary Material

gkaf392_Supplemental_File

## Data Availability

The web server is available at https://asoptimizer.s-core.ai. This website is free and open to all users and there is no login requirement. Source code is available at https://github.com/Spidercores/ASOptimizer (GitHub) and https://doi.org/10.6084/m9.figshare.28829144 (Figshare).
