# Supplementary material for: ASOptimizer: optimizing chemical diversity of antisense oligonucleotides through deep learning
Source: Nucleic Acids Res. 2025 May 16;53(W1):W39–44. doi: 10.1093/nar/gkaf392 (PMC12230697; doi:10.1093/nar/gkaf392)
Supplement: gkaf392_Supplemental_File [file gkaf392_supplemental_file.pdf]

Supplementary Material for ASOptimizer: Optimizing Chemical Diversity of Antisense Oligonucleotides through Deep Learning

Hyperparameters related to the model architecture and training

Table S1: A model-parameters used in our ASOptimizer.

Type	Hyperparameters
EGT-specific parameters	Max length (N): $\{\mathbf{516}\}$<br>Node embedding dimension (d_h): $\{\mathbf{768}\}$<br>Edge embedding dimension (d_H): $\{\mathbf{128}\}$<br># of transformer layers (L): $\{\mathbf{12}\}$<br># heads in the multi-head attention: $\{\mathbf{32}\}$<br># virtual nodes: $\{\mathbf{8}\}$<br>Maskoff probability in random attential masking: $\{\mathbf{0.1}\}$<br># of fully-connected (FC) layers: $\{\mathbf{2}\}$<br>Embedding dimension in FC layers: $\{\mathbf{768}\}$
Other generic training parameters	Activation function: $\{\textbf{Elu}\}$<br>Learning rate: $\{\mathbf{10^{-5}}\}$<br>Regularization strength: $\{\mathbf{10^{-2}}\}$

Further validation of ASOptimizer

We further validated *ASOptimizer*'s performance across a range of base sequences. Figure S1 illustrates sequence-wise performance on the test dataset, consisting of 46 unique sequences sorted by Pearson correlation. To ensure reliability, we filtered sequences to include only those with more than five experimental points per sequence. The results show that over half of the sequences demonstrated a remarkable correlation, exceeding 0.5. The Pearson correlation between the predicted and in-vitro inhibitions is shown, with a weighted mean of $\rho = 0.582$ (where the weights correspond to the number of experimental points per sequence) and a standard deviation of 0.292. See the supplementary material for detailed information and performance for each sequence.

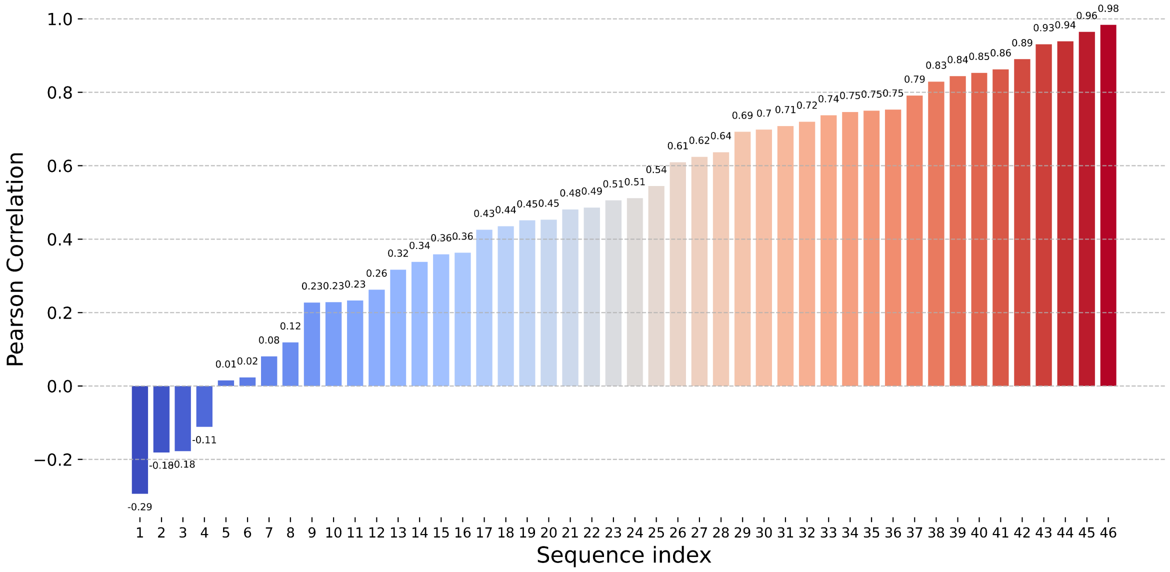

Figure S1: In silico validation of the chemical engineering module. Sequence-wise performance on 46 unique test sequences, sorted by Pearson correlation. The values above the bars represent the sequence-wise correlation coefficients.
